# Diagnostic performance of chest X-ray for the diagnosis of community acquired pneumonia in acute admitted patients with respiratory symptoms

**DOI:** 10.1186/1757-7241-21-S2-A21

**Published:** 2013-09-09

**Authors:** Christian B Laursen, Erik Sloth, Jess Lambrechtsen, Annmarie Touborg Lassen, Poul Henning Madsen, Daniel Pilsgaard Henriksen, Jesper Rømhild Davidsen, Finn Rasmussen

**Affiliations:** 1 Department of Respiratory Medicine, Odense University Hospital, Denmark; 2 Department of Anaesthesia and Intensive Care, Aarhus University Hospital, Skejby, Denmark; 3 Department of Medicine, Odense University Hospital - Svendborg, Denmark; 4 Medical Emergency Department, Odense University Hospital, Denmark; 5 Department of Medicine, Sygehus Lillebælt, Denmark; 6 Department of Allergy and Respiratory Medicine, Near East University Hospital, Nicosia. North Cyprus, Mersin 10, Turkey

## Background

Despite being a routine diagnostic modality for the diagnosis of community acquired pneumonia (CAP), few studies have evaluated the diagnostic performance for the diagnosis of CAP according to an initial performed chest x-ray (CXR) in an emergency department (ED). As a part of a prospective observational study of patients admitted with acute respiratory symptoms in an ED, this relation was evaluated.

## Methods

A prospective cross sectional observational study was conducted in a medical ED. Patients were included if one or more of the following clinical findings or symptoms were present: respiratory rate > 20/minute, oxygen saturation < 95 %, oxygen therapy initiated, dyspnoea, cough, or chest pain. The assessments of the CXR by the treating physician in the ED and the radiologist were prospectively registered. Blinded audit by three physicians who used predefined diagnostic criteria was used as gold standard.

## Results

342 patients were screened of whom 139 (40.6%) were included. An acute CXR was performed in 121 (87.1%) of the patients. In 50 (41.3%) of the patients, the treating physician in the ED described the CXR with opacity due to CAP. The radiologist described opacity due to CAP in 54 (44.3%) of the cases. Audit found 58 (47.9%) of the patients met the predefined criteria for CAP. Diagnostic performance of the CXR evaluated by the treating physician was: sensitivity 70.7% (95%CI 57.3-81.9%), specificity 85.7% (95%CI 74.6-93.3%), PPV 82.0% (95%CI 68.6-91.4%), NPV 76.1% (95%CI 64-5-85.4%) and ROC area 0.782 (95%CI 0.709-0.855%). Diagnostic performance of the initial CXR evaluated by a radiologist was: sensitivity 69.0% (95%CI 55.5-80.5%), specificity 77.8% (95% CI 65.5-87.3%), PPV 74.1% (95%CI 60.3-85.0%), NPV 73.1% (95%CI 60.9-83.2%), and ROC area 0.734 (95%CI 0.654-0.813). The overall agreement between the treating physician and radiologist was 71.4% (ê 0.429).

## Conclusion

Based on these findings, the initial CXR will only be able to diagnose seven out of ten patients with CAP in patients with respiratory symptoms who are acutely admitted to a medical ED. In accordance with Fleiss’ guidelines, the agreement between treating physician and radiologist for the assessment of chest x-ray for the diagnosis of CAP is fair to good.

